# WHAT PANDEMIC RECOVERY WILL LOOK LIKE FOR OLDER, MULTICULTURAL WORKERS

**DOI:** 10.1093/geroni/igac059.1016

**Published:** 2022-12-20

**Authors:** Cassandra Burton, Aisha Cozad, Katherine Bridges

**Affiliations:** AARP, Atlanta, Georgia, United States; AARP, Washington, District of Columbia, United States; AARP, Deer Isle, Maine, United States

## Abstract

AARP has a long history of being an advocate for marginalized and vulnerable adults. AARP staff will discuss policy needs and what the post pandemic workplaces needs to ensure that older LGBTQ people can thrive in the workplace with dignity and respect. The 2021 AARP’s Vital Voices research will be used to showcase the economic impact the pandemic has had on older adults, African American communities, Hispanic/Latino communities, Asian Pacific Islander communities and LGBTQ communities. AARP staff will discuss strategies and tactics needed to ensure that opportunities for economic recovery for older adults. The survey gathers information to gauge opinions on a range of topics as well as breaking and current issues. Among other issues, it explores concerns about racism and experience with age discrimination and employment experience among older adults.

